# Supplementary material for: The In Vitro Contractile Response of Canine Pregnant Myometrium to Oxytocin and Denaverine Hydrochloride
Source: Biology (Basel). 2023 Jun 15;12(6):860. doi: 10.3390/biology12060860 (PMC10295642; doi:10.3390/biology12060860)
Supplement: Supplementary file 1 [file biology-12-00860-s001.zip › biology-2405965-supplementary.pdf]

Table S1. Detailed information about the bitches including breed, serum progesterone concentration at the examination before surgery (P4; in ng/mL), age (in years), body weight (in kg), the total number of all puppies in the litter, and the reason for the C-section (PUI = primary uterine inertia; SUI = secondary uterine inertia). Reasons for elective C-section were breed of the bitch, singleton pregnancy, or concomitant uterine infection.

Breed.	P4 (ng/mL)	Age (years)	Body weight (kg)	Total number of puppies	Reason for C-section
Miniature Bull Terrier	10.8	2.5	13.4	2	Elective C-section
Miniature Bull Terrier	1.9	No data	11.8	3	Obstruction
Labrador Retriever	*	2.5	29	11	SUI
Labrador Retriever	*	5	35.4	7	SUI
Bull Terrier	18.6		14.8	3	SUI
German Shepherd	*	5	34.5	7	PUI
French Bulldog	0.7	5.5	10	5	PUI
Great Swiss Mountain Dog	4.8	4.5	49.7	4	Elective C-section
Pomeranian	2.6	4.5	6.5	2	PUI
Leonberger	1.0	3	69	12	SUI
Cairn Terrier	13.9	4.5	9.1	1	Elective C-section
Golden Retriever	1.8	6.8	32.5	8	PUI
Pomeranian	21.9	5.25	5.6	1	Elective C-section
German Shepherd	*	5.25	32.5	1	PUI
Chihuahua	*	7.25	2.5	2	SUI
Miniature Bull Terrier	2.3	No data	14.2	5	Elective C-section
French Bulldog	*	2	12.4	6	Obstruction
Bolonka Zwetna	3.1	6	4.7	1	PUI
Briard	19.9	4	33.6	1	Elective C-section
Australian Shepherd	2.1	9	20.0	6	PUI
Sheltie	5.0	8.5	13.2	8	SUI
Staffordshire Bullterrier	0.9	5.5	12.8	8	SUI
Boston Terrier	13.7	6	9.4	1	Elective C-section
Golden Retriever	5.0	4	38.2	6	PUI
Miniature Bull Terrier	2.5	4.8	17.8	3	Elective C-section

* not available
